# Prehospital emergency medical technicians can perform ultrasonography and blood analysis in prehospital evaluation of patients with chronic obstructive pulmonary disease: a feasibility study

**DOI:** 10.1186/s12913-021-06305-7

**Published:** 2021-03-31

**Authors:** Giti Nadim, Christian B. Laursen, Pia I. Pietersen, Daniel Wittrock, Michael K. Sørensen, Lars B. Nielsen, Claus-Henrik Rasmussen, Helle Marie Christensen, Simon Helmerik, Gitte Jørgensen, Ingrid L. Titlestad, Annmarie T. Lassen, Søren Mikkelsen

**Affiliations:** 1 Emergency Medicine Research Unit, Odense University Hospital, Odense, Denmark; 2 Department of Clinical Research, Odense Respiratory Research Unit (ODIN), University of Southern Denmark, Odense, Denmark; 3 Department of Respiratory Medicine, Odense University Hospital, Odense, Denmark; 4 Ambulance Syd, Odense, Denmark; 5 Responce & Falck Denmark, Kolding, Denmark; 6 Department of Health Planning, Prehospital Services, Region of Southern Denmark, Vejle, Denmark; 7 The Prehospital Research Unit, Region of Southern Denmark, Odense University Hospital, Odense, Denmark; 8 Department of Aneaesthesiology and Intensive Care Medicine, Mobile Emergency Care Unit, Odense University Hospital, Odense, Denmark

**Keywords:** Emergency medical technicians, Point of care; ultrasound, Chronic pulmonary obstructive disease

## Abstract

**Introduction:**

Crowding of the emergency departments is an increasing problem. Many patients with an exacerbation of chronic obstructive pulmonary disease (COPD) are often treated in the emergency departments for a very short period before discharged to their homes. It is possible that this treatment could take place in the patients’ homes with sufficient diagnostics supporting the treatment.

In an effort to keep the diagnostics and treatment of some of these patients in their homes and thus to reduce the patient load at the emergency departments, we implemented a prehospital treat-and-release strategy based on ultrasonography and blood testing performed by emergency medical technicians (EMT) or paramedics (PM) in patients with acute exacerbation of COPD.

**Method:**

EMTs and PMs were enrolled in a six-hour educational program covering ultrasonography of the lungs and point of care blood tests. During the seasonal peak of COPD exacerbations (October 2018 – May 2019) all patients who were treated by the ambulance crews for respiratory insufficiency were screened in the ambulances. If the patient had uncomplicated COPD not requiring immediate transport to the hospital, ultrasonographic examination of the lungs, measurements of C-reactive protein and venous blood gases analyses were performed. The response to the initial treatment and the results obtained were discussed via telemedical consultation with a prehospital anaesthesiologist who then decided to either release the patient at the scene or to have the patient transported to the hospital. The primary outcome was strategy feasibility.

**Results:**

We included 100 EMTs and PMs in the study. During the study period, 771 patients with respiratory insufficiency were screened. Uncomplicated COPD was rare as only 41patients were treated according to the treat-and-release strategy. Twenty of these patients (49%) were released at the scene. In further ten patients, technical problems were encountered hindering release at the scene.

**Conclusion:**

In a few selected patients with suspected acute exacerbations of COPD, it was technically and organisationally feasible for EMTs and PMs to perform prehospital POCT-ultrasound and laboratory testing and release the patients following treatment. None of the patients released at the scene requested a secondary ambulance within the first 48 h following the intervention.

## Background

The emergency medical system (EMS) is a critical first link in the chain of survival and has the capacity for the early management of acutely ill patients [1–3].

The usual approach: “*To transport the patient to the hospital*”, where all acutely ill patients are consistently transported to hospital for further care is challenged by increasing costs because of an ageing population, the increased incidence of chronic diseases, the socio-economic disparity associated with most chronic diseases and the ensuing crowding of the emergency departments [4, 5].

Patients with acute exacerbations in chronic obstructive pulmonary disease (AE-COPD) are frequently transported to the emergency room of the hospital. In many cases, these patients are discharged shortly after admission following a brief treatment consisting of broncholytics and in some cases corticosteroids and antibiotics [6, 7]. The transport of these patients for very short stays at the hospital have both implications for health service costs as well as for the patients’ well-being. Among prehospital providers, there is a notion that many of these patients are not really interested in the short stays at the hospital, but would rather stay home if this decision could be supported medically. Could these short term stay be reduced, the total health care costs may be reduced. As some of the patients with AE-COPD can be efficiently treated with bronchodilation, steroids and in some cases antibiotics there is a potential room for prehospital evaluation and treatment in the homes of these patients without the a need for transportation to a hospital [8, 9].

A treat and release strategy, however, requires that life-threatening differential diagnoses, such as pneumothorax, pleural effusion or cardiogenic pulmonary oedema, have been ruled out before deciding not to transport the patient to the hospital and instead initiate goal-directed AE-COPD treatment in the patients home.

Point of care ultrasound (POCUS) of the lungs have previously been demonstrated to have a high diagnostic accuracy regarding pulmonary interstitial syndrome as well as pneumothorax [10]. Both of these diagnoses are important differential diagnosis in evaluation of patients with suspected AE-COPD and are easy for non-experienced POCUS operators to learn to identify [10].

We designed a study to investigate whether a treat-and-release strategy in COPD patients was feasible if carried out by the emergency medical technicians (EMT) and paramedics (PM). The strategy was based on clinical assessment and standard broncholytic therapy supported by the use of POCUS of the lungs, measurements of venous blood gases, and blood tests to evaluate the inflammation level. All of these investigations were performed by the EMT or the PM at the scene. Following the clinical and paraclinical investigations, telemedicine counselling between the EMTs or PMs and a prehospital anaestesiologist clarified whether the patient was in a condition that allowed for the patient to be released at the prehospital scene. If indicated, the EMT or PM could initiate oral treatment with steroids and/or antibiotics and refer the patient to the general practitioner the following day.

The aim of the study was to test the technical setup, the clinical training of the personnel, and the implementation and feasibility of the treat-and-release strategy.

## Material and methods

This is a descriptive study of the technical setup and the clinical training of the personnel, and the implementation and feasibility of the strategy.

### System setting

In Denmark, five regional emergency medical dispatch centres handle all healthcare related calls and dispatches the relevant prehospital response units based on the perceived urgency of the health-related problem [11]. The EMS in the Region of Southern Denmark is a three tiered system consisting of approximately 70 ambulances, three paramedic-manned rapid response vehicles, and six ground-based anaesthesiologist-manned mobile emergency care units (MECU) [12, 13].

The regional EMS and the private ambulance entrepreneur employ approximately 600 EMTs and PMs. The basic education for an EMT has a duration of 1 year and is carried out within the public health educational system. However, at least one of the two mandatory EMTs manning an ambulance must have received supplemental education consisting of an additional 18 months of internship in an ambulance service and a further 5 weeks of education. An EMT may continue the education and may become a PM after 3 years of practice as an EMT and having undergone a further 5 weeks of theoretical and practical education [14]. Furthermore, paramedics are obligated to participate in at least 1 week of supplemental training / continuous education per year. All ambulance personnel work by delegation from a physician, and handle medications independently. In the Region of Southern Denmark, the EMTs and PMs are usually designated to one ambulance station only. The EMTs work in two different shifts: One shift consisting of 12-h rotas and one shift consisting of 24-h rotas. Interhospital transports of patients are usually carried out by the ambulance personnel working in the 12-h rotas while personnel working in the 24 h rotas to a larger extent handle the emergency calls. Personnel working in 24-h rotas thus in principle have more contacts with the acutely ill patients. As there are also differences in the workload between the urban ambulance stations and the rural ambulance stations, the number of emergency missions carried out by each individual EMT or PM differs considerably.

### Study participants

The task of training all the EMTs and PMs (i.e. 600 people) in the RSD was considered unfeasible. Furthermore, the number of patient contacts in a given observation period depends on both the rotas and the particular ambulance station that each EMT or PM was attached to. We thus restricted the study participants to the ambulance personnel operating the 24-h rotas in the five ambulance stations in the region with the highest number of patient contacts. This decision was considered the optimal number of EMTs and PMs in relation to the possibility of encountering acutely ill COPD patients in the study period. The strategy resulted in a total of 100 EMTs or PMs participating in the educational program.

### Eligible patients

The study was conducted from October 1st 2018 – May 31st 2019, the months of peak seasonal occurrence of exacerbations in chronic obstructive pulmonary disease. During the study period, the EMTs or PMs were instructed to screen all patients calling for an ambulance because of dyspnea. Patients were enrolled if all of the conditions listed below were present.

### Inclusion criteria

- Dyspnea as sole complaint in a patient that could inform the EMT or PM that he/she had previously been diagnosed with COPD.
- Based on clinical judgment, the patient did not require immediate transport to the hospital (e.g. a “scoop and run strategy” was not indicated).
- According to the EMTs or PMs “clinical judgment”, the patient was assessed as having a potential for being released at the scene following treatment.
- Based on the patient’s history, clinical examination, the vital parameters, and the ECG, there was no reason for the EMT or PM *not* to assume that the condition was caused by COPD.
- The patient was able to fully comprehend the nature of the investigations and the extent of the treatment.
- The patient being able to self-administer any steroid or antibiotic treatment initiated by the EMT or PM at the scene.

### Exclusion criteria

- EMT or PM not educated in the use of the equipment.
- Patients in which COPD was not the main complaint (e.g. chest pain, thoracic trauma).
- Patients that were unable to understand the intervention (e.g. language barriers, dementia).

### Basic examinations

The EMTs or PMs performed an intial screening examination of the patients including vital parameters, ECG, and pulmonary auscultation before considering inclusion of the patient into the protocol.

### The equipment

#### Portable smartphone ultrasound

**Fig. 1:**
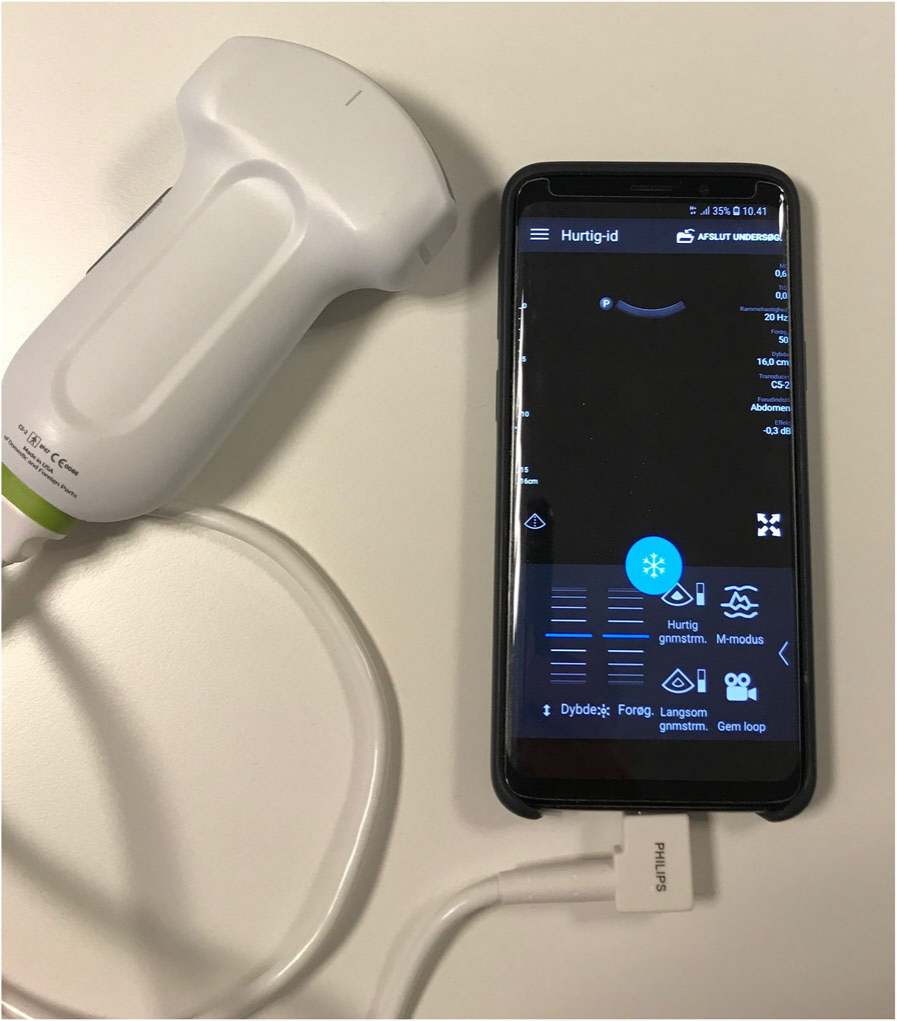
Lumify portable ultrasound device

Lumify (Lumify C5–2, Philips Eindhoven, The Netherlands) is a portable ultrasound device applicable to a smartphone and other handheld devices. See Fig. 1. The device consists of three different transducers (high frequency linear tranducer, curved low frequency abdominal transducer, low frequency cardiac transducer) and cables, a smartphone (Galaxy G960 S9, Samsung, Seoul, South Korea) with an installed Lumify-app and a power cord. The system is powered by React’s collaborative platform (React-Secure-app, Innovative Imaging Technologies Inc., Philips, Eindhoven, The Netherlands) which provides secure instant messaging/file transfer, interactive video conferencing, and real-time integrated tele-ultrasound (i.e. these functions require that the mobile phone receiving the call has an installed React-Secure-app). For a real-time tele-ultrasound or tele-medical video supervision, the supervising units and the MECU physicians used an Apple iPhone 8, 64GB (Apple, Cupertino, California, USA) equipped with a React-Secure app.

##### POCUS protocol and diagnostic criteria

We applied a specific prehospital POCUS protocol in which only the anterior and lateral scanning zones on each hemithorax are examined. This protocol is a simplified version of a protocol adapted for in-hospital use and the protocol has previously been used and validated prehospitally in patients with respiratory failure [10].

The protocol was used in a focused manner addressing the following dichotomous yes/no questions:

- Pneumothorax present?
- Pleural effusion present?
- Interstitial syndrome present?
- Lung consolidation present?
- Other obvious abnormal finding present?

The diagnostic criteria used for pneumothorax, pleural effusion, and lung consolidation was in accordance with criteria recommended in international consensus statements [15]. The diagnostic criteria used for interstitial syndrome were in accordance with criteria previously validated in a prehospital setting [10].

#### QuikRead GO

The QuikRead Go (QuikRead go CRP instrument, Medic Denmark, Brøndby, Denmark) analyses the quantitative measures of c-reactive protein (CRP) in whole blood and plasma. The system consists of a photometer, which is designed and calibrated for both photometric and turbidimetric measurements and a ready-to-use reagent kit.

CRP is an acute-phase protein synthesized by the liver within 6–8 h of inflammation. CRP is a valuable biomarker in differentiating between viral and bacterial infection. Furthermore, CRP performs better in predicting bacterial infections than traditional biomarkers, such as white blood cell count and absolute neutrophil count [16]. While standard laboratory CRP results are available within 60–90 min of sampling, CRP test results with QuikRead Go are available within 2 min of sampling, with values ranging from 5 to 200 mg/L (values lower than 5 are shown as < 5 mg/L and values greater than 200 as > 200 mg/L). The apparatus cannot function in temperatures lower than 15 °C.

These assets makes the use of point-of-care CRP at the emergency departments (ED) or prehospital settings relevant [17].

#### I-STAT

The i-STAT (i-STAT Alinity, Abbott, Illinois, USA) is a point-of-care (POC) analyser that measures blood gases and electrolytes with ion-selective electrode potentiometry. The handheld device operates with single-use test cartridges (i-STAT Alinity Base station, Abbott), requires the application of two to three drops of blood in the cartridge, and can deliver results within minutes [18]. The system requires a temperature above 15 °C to operate [19].

### Education and training of the EMTs and PMs

The eligible EMTs and PMs each completed one study session held within small groups. The lessons were constructed as didactic lectures covering basic ultrasound scanning technics, normal ultrasound anatomy, imaging interpretation of both normal and pathologic lung (i.e. sonographic signs, including ‘lung sliding’, ‘B-lines’, ‘the lung point sign, pleura effusion, and consolidation) [15, 20]. Following the didactic lectures, practical physician-supervised ultrasound sessions were held in which the participants scanned each other. By the end and to ensure competence, the participants demonstrated their knowledge and skills to the supervisor by receiving a case history, performing an examination, and interpreted ultrasound clips (normal and pathological) presented on a laptop.

The aim of the lessons were to enable the participants to scan the four anterolateral zones of the lungs (corresponding to zone 1 and 3 in the focused lung ultrasound (FLUS) protocol) using a curved low frequency abdominal transducer [21]. Following the course, the participants should be able to recognise basic pulmonary pathology signs, and to save the ultrasound loop-images for later assessment and evaluation.

In total, the EMTs and PMs received 6 h of didactic lectures and supervised hands-on training. Furthermore, the participants received two lessons concerning the use of POC equipment intended for blood testing. In order to ensure standardised use of the POC-equipment and to avoid procedure errors, a detailed protocol containing *how-to-use manuals* for the devices and described *when-to-use indications* could be accessed by the participants in the internal educational internet-based system. As a quality assurance measure, the stored ultrasound clips were continuously checked during the study by two of the investigators (CBL, PIP).

### Intervention

The EMTs and PMs in the interventional ambulances were equipped with 1) a QuikRead GO apparatus for measuring c-reactive protein, 2) an i-Stat device for measuring venous blood gases (including electrolytes), and 3) a portable ultrasound-scanning device (Lumify, Phillips) for the examination of the lungs.

When dispatched to COPD-patients with respiratory insufficiency, in addition to the standard treatment, the interventional-ambulances would 1) collect blood analysis for c-reactive protein and blood gases from an antecubital vein, and 2) perform a focused ultrasound-scan of the lungs according to the standardized protocol. In all cases, the intervention crew could transmit the ultrasound loop-images in real time to the anesthesiologist manning a nearby MECU.

If the stabilising treatment applied on-site significantly improved the condition of the patient, the clinical findings were discussed over the telephone with the nearest available MECU physician. The telemedical conference between the interventional EMT/PM and the MECU-physician would clarify whether the clinical condition of the patient after initiated treatment and the obtained point-of-care test results would permit the release of the patient at home. This decision was made without prior establishing of guidelines or rules. Patients released at home were instructed to contact their primary care physician the next weekday. Should the diagnostic findings indicate a need for treatment with corticoids and/or empirical antibiotics, the patient was given a three-day course of methylprednisolone and/or amoxicillin combined with clavulanic acid according to current guidelines. Furthermore, the patient was urged to contact the emergency medical dispatch centres in case of worsening of the symptoms. Finally, the patient was handed a written report, including the clinical and laboratory findings, and the treatment given onsite. This report was intended for the patient’s general practitioner.

All investigations pertaining to the patient inclusive the ultrasonographic findings were documented in the prehospital electronic journal registry.

**Fig. 2:**
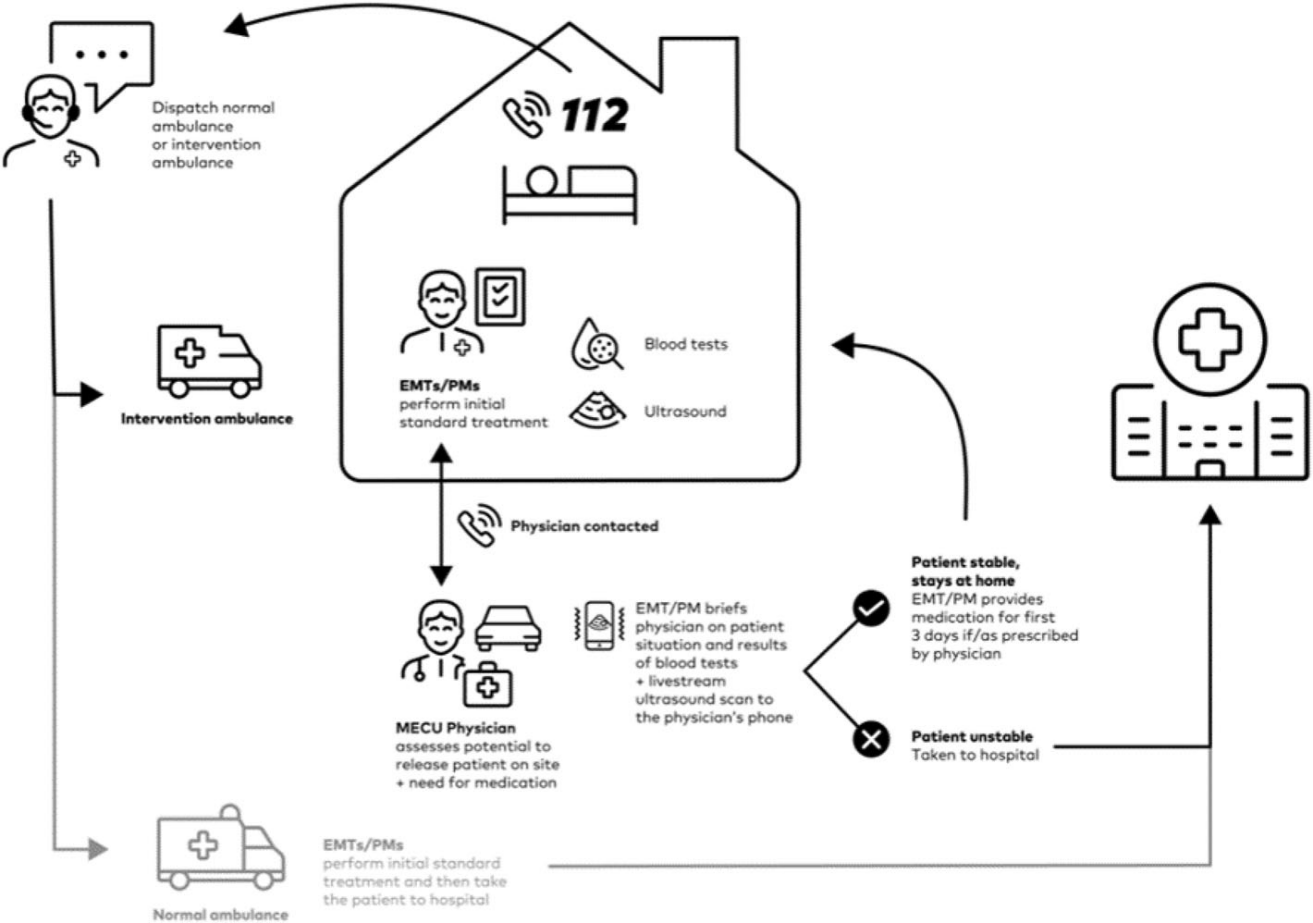
Overview of study procedures

See Fig. 2 (Study overview) for study procedures.

### Data management and statistical analyses

Data were retrieved from the national Danish Prehospital Medical Records Database [12].

Demographic data are presented as median and quartiles or range (where appropriate). All data were analysed using non-parametric statistics (Kruskal-Wallis test). Differences were considered significant when *p* < 0.05. All data and tables were categorised and prepared using Microsoft Office Excel 2016 (Microsoft Corporation, Redmond, Washington, USA). All statistical calculations were performed using STATA 16.1 (StataCorp, College Station,Texas, USA).

### Research ethics

The study was conducted in compliance with all national regulations governing the protection and privacy of human subjects and the Helsinki Declaration. Informed consent was obtained from the participants when they were included in the study. Scientific ethical approval for this study was waived by the Regional Scientific Ethics Committee of Southern Denmark as the committee considered the study a quality assurance study (project-ID: S-20182000 − 130). According to the Danish legislative requirements, the study was subsequently approved as a quality assurance study by the Prehospital Director of the Region of Southern Denmark (project-ID: 19/14433) and approved by the Danish Data Protection Agency (project-ID: 20/24845). Before initiating the study an agreement was made regarding ownership of data and interpretation of data. The agreement stated that the academic collaborators who had no commercial or other interest in either of the participating organisations (authors GN, CBL, PIP, HMC, ILT, ATL, and SM), were responsible for analyses, interpretation of data, and for drafting the first version of the manuscript. Furthermore, these authors had the full authority to decide if and where to seek to publish the results.

## Results

The education and training of the 100 EMTs and PMs in the use of the point-of-care blood analysis apparatuses and performing POCUS of the lungs was carried out in the 3 months preceding the peak seasonal occurrence of COPD.

During the 8 months of the study period, from October 1st 2018 to May 31st 2019, an ambulance from the five ambulance stations in question was dispatched to 771 patients in respiratory distress. The medical records of all these patients were audited post hoc by one author (SM).

For various caused (COPD not singular reason for calling an ambulance; emergency transportation with lights and sirens required; substitute EMT operating the ambulance), only 81 patients were considered eligible to enter the study.

### Inclusion of patients and outcome of the intervention

Of the 81 potentially eligible patients, 41 were included in the study. Of these 41 patients, 20 patients (49%) were released at the scene following treatment. In ten of the 41 cases, technical failure of the equipment (primarily related to blood tests) led to a patient’s exclusion from the study. Eleven patients were transported to hospital following on-scene treatment. Of these, five patients were admitted to hospital by a MECU following telemedical consultation, four were admitted at the discretion of the EMT/PM, and two were admitted due to patient’s request following the intervention.

### For details, see Fig. 3 (flowchart)

The overall median age of all the included patients was 70 years (quartiles 65–77 years) and 53.7% were females. Twenty patients were released at the scene following treatment; three of these despite the examination program not being fully completed. The patients released at the scene had a median age of 70 years (61, 73 years) while patients admitted to hospital for all reasons had a median age of 73 (66, 81 years). This difference was not significant (*p* = 0.136). For demographic overview and results obtained through POC-testing, see Table 1. Of the 20 patients that were released at the scene, nine patients were prescribed corticosteroids following consultations between the EMT or PM and the physician. A further three patients received corticosteroids prior to the incident. Six of the 20 patients that were released were given antibiotics by the EMTs/PMs prior to release. A further two patients already received antibiotics when they called the ambulance.

**Fig. 3:**
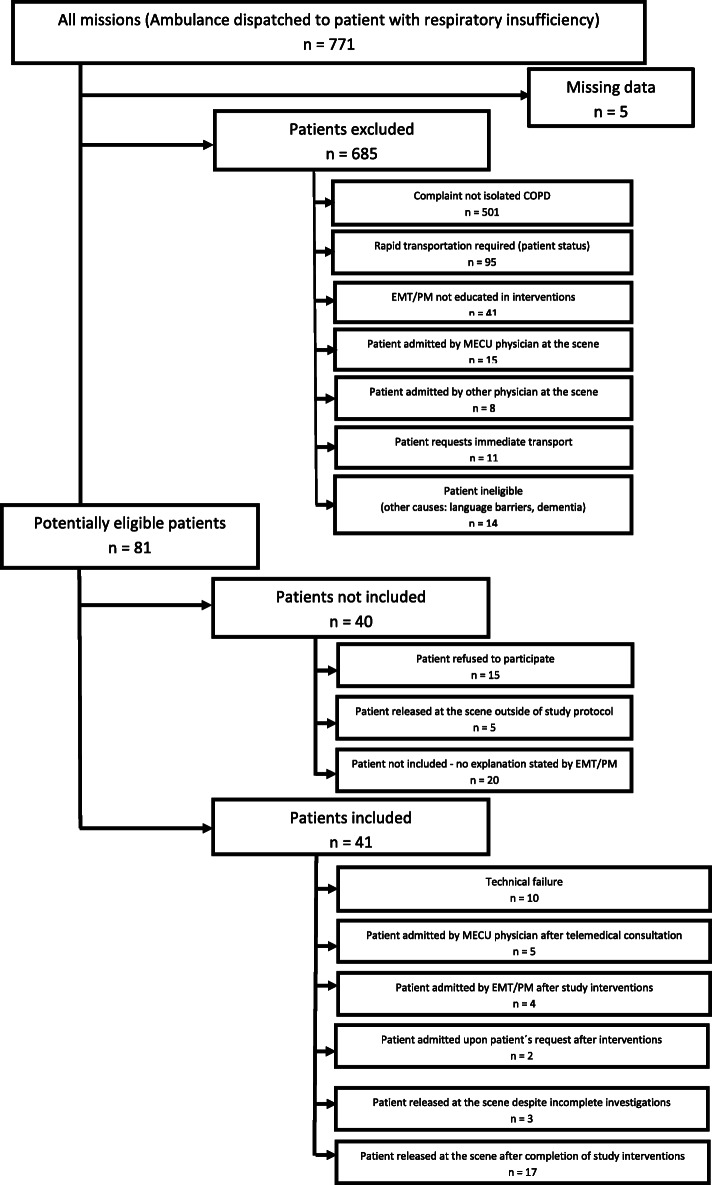
Flow diagram of the total population of patients. Study intervention was considered complete when pulmonary sonography was recorded, venous blood gases and C-reactive protein was assessed

**Table 1:** Overview of the demographics and point-of-care blood tests for the patients included in the study

Outcome	Age	Sex	pH	pCO2 (kPa)	pO2 (kPa)	HCO3 (mmol/l)	Base Excess	Glucose (mmol/l)	C-reactive protein
Patient released at the scene	69	F	7.37	8.3	6.9	35.7	10	7.3	12
	65	F	7.39	5.5	5.2	25.0	0	4.8	3,1
	88	F	7.37	8.0	n/a	n/a	n/a	n/a	1,2
	72	M	7.38	5.4	5.9	24.1	−1	10.0	17
	54	F	7.40	6.6	4.2	30.3	5	9.5	9,4
	70	M	7.27	9.0	3.4	31.3	4	6.3	0,8
	64	M	7.37	6.6	3.1	28.6	3	10.2	64
	61	M	7.33	6.0	3.1	23.9	-2	6.0	6,3
	76	M	7.37	5.6	4.2	23.9	-1	5.4	8,7
	72	M	7.36	7.0	4.4	29.7	4	6.2	7,6
	73	F	7.36	11.8	7.7	49.7	24	11.1	5,7
	69	F	7.30	8.7	5.7	32.4	6	6.4	1,5
	58	F	7.43	5.7	2.8	28.1	4	5.9	1,1
	74	M	7.33	9.0	3.3	35.4	9	7.1	6,4
	77	F	7.42	4.6	4.6	22.7	-2	5.0	52
	71	F	7.43	4.6	4.7	22.9	-1	7.1	43
	71	M	7.36	6.3	2.4	26.8	1	6.3	161
	66	M	7.40	7.3	4.0	33.6	9	7.7	n/a
	46	M	7.28	13.6	2.9	48.5	22	6.4	n/a
	50	F	n/a	n/a	n/a	n/a	n/a	n/a	31
Technical failure – patient admitted to hospital	84	M	n/a	n/a	n/a	n/a	n/a	n/a	17
	66	F	n/a	n/a	n/a	n/a	n/a	n/a	n/a
	70	M	n/a	n/a	n/a	n/a	n/a	n/a	141
	48	M	n/a	n/a	n/a	n/a	n/a	n/a	n/a
	52	F	n/a	n/a	n/a	n/a	n/a	n/a	n/a
	77	F	n/a	n/a	n/a	n/a	n/a	n/a	n/a
	81	F	n/a	n/a	n/a	n/a	n/a	n/a	n/a
	76	M	7.28	5.8	4.5	20.7	−6	5.6	54
	70	F	7.32	6.7	2.3	26.1	0	8.8	n/a
	79	F	7.36	5.7	9.0	24.3	−1	11.6	20
Patient admitted to hospital by EMT	66	F	7.27	10.9	3.7	37.6	11	6.0	41
	82	M	7.29	8.5	3.5	30.6	4	n/a	3,8
	52	F	7.43	6.3	6.5	31.4	7	8.3	66
	87	F	7.37	6.4	4.5	27.9	3	7.5	33
Patient admitted to hospital by physician	85	M	7.31	9.0	n/a	n/a	n/a	n/a	18
	79	M	7.29	9.5	3.3	34.6	8	4.9	n/a
	77	M	n/a	n/a	n/a	n/a	n/a	n/a	33
	66	M	n/a	n/a	n/a	n/a	n/a	n/a	n/a
	57	F	n/a	n/a	n/a	n/a	n/a	n/a	n/a
Admitted to hospital on patient’s request	66	F	7.35	7.3	9.9	30.2	5	6.5	4,9
	81	F	7.38	7.6	4.5	34.0	9	5.6	17

None of the patients released at the scene requested a renewed ambulance within the first 48 h following the intervention.

### On-scene time

The on-scene time (time from arrival at the address and until the ambulance left the address) spent by the ambulances differed when treating patients that although assessed as potentially eligible, for some reason were not included into the study, and patients that were included in the study. When tending to potentially eligible patients that were not included in the study, the ambulance spent 18 min (quartiles 8–22 min) at the scene, while they stayed at the scene for 70 min (quartiles 44–89.5 min) when including patients in the study. This difference was significant *p* < 0.0001.

## Discussion

In this study, we have found that in selected cases of acute exacerbations of COPD, a treat-and-release strategy can be feasible when driven by EMTs and PMs with access to telemedical consultation with prehospital anaesthesiologists.

General home care of patients with COPD is already an established possibility in some countries [22]. It has further been reported that the majority of acute COPD patients receiving care in the EDs are discharged within 24 h of the admission [6, 7]. A logical notion thus could be that patients with acute exacerbations of COPD also to some extent could be treated in their homes. In Denmark, home care of patients with COPD usually involves nurses and the patients’general practitioner. However, at present, there are no firm recommendations available about which patients with an exacerbation are most suitable for Hospital-at-Home or early discharge [22].

Acute respiratory insufficiency in a patient with known COPD is usually caused by an exacerbation of the underlying illness. In clinical practice, however, other aetiologies present with the same symptoms as acute exacerbation of COPD, and upfront diagnostics are essential to establish the diagnosis and start the correct treatment [23, 24]. A prerequisite for treating and releasing the patient out of hospital is thus an assurance that what the care giver perceives as an exacerbation of COPD is not, in essence, a concurring bout of a potentially life threatening condition. To that end, the exclusion of pneumothorax and cardiogenic pulmonary oedema which in most instances requires admission to hospital, is paramount.

Although the use of POCUS and blood gas analyses are routine examinations in most EDs and emergency care units today, the systematic use of this technology in the prehospital field is limited.

Other studies have reported the use of POCUS in various healthcare settings, including use by prehospital physicians, EMTs, flight nurses, and emergency physicians in military combat and on both ground and air ambulances [25–27]. Furthermore, other researchers have found that an accurate interpretation of POCUS images by prehospital physicians and non-physicians may lead to specific interventions or may facilitate the hospital preparations before the arrival of the patient [28–31]. Even though POCUS have been demonstrated to have a high diagnostic accuracy for diagnosing pleural effusion, pneumothorax, and interstitial syndrome (e.g. cardiogenic pulmonary oedema), it still have some limitations [20]. In COPD patients with suspected pneumonia, an important differential diagnosis is community acquired pneumonia. The ability of POCUS to visualise lung consolidation as part of a pneumonia is limited as pathological findings in lung parenchymal that does not lie directly in contact with the visceral pleura cannot be visualised by ultrasonography. Despite this limitation, studies have indicated that the diagnostic accuracy of POCUS is acceptable and not inferior to chest X-ray [32]. POCUS findings should, however, always be critically appraised and integrated with the findings and results of the entire diagnostic process. Our combination of POCUS and blood analyses thus allowed for an improved on scene diagnostic process regarding the possible aetiology of the acute exacerbation of COPD. The CRP measurements and the blood gas analyses assisted the telemedical advisor in deciding whether the EMTs or PMs at the scene should initiate antibiotic therapy. Furthermore, the POCUS assisted in the decision making process as the ultrasound examination relayed to the physician, together with the verbal description of the POCUS findings assisted in ruling out other potentially life threatening causes of respiratory insufficiency.

The treat-and-release strategy reduced the time from the occurrence of symptoms to first treatment. The strategy also helped to distinguish between patients requiring admission to hospital and patients with mild symptoms who could be treated at home.

The median time needed to complete the intervention on the scene was 70 min. Thus, the ambulances spent longer time at the scene than ambulances simply loading the patients and heading for hospital. In some prehospital cases, reducing the time spent at the scene is important. This is, for example the case for trauma patients, where longer on scene time is associated with adverse prognosis [33, 34]. However, it is important to underline, that scene time was prolonged for only the stable COPD patients who were included in the intervention while unstable patients were transported to hospital immediately. It should be noted, however, that prolonged on-scene time may reduce the general availability of ambulances potentially influencing “the next patient”.

The adequate quality of ultrasound images obtained in our study suggests that the EMTs and PMs had sufficient POCUS abilities to perform and obtain the predefined views in the used protocol. In our study, the ambulance crews received 6 h of training. This constituted a more elaborated training course than in other studies. Teaching lessons of down to 1 h has been reported to enable EMTs to interpret POCUS images for specific life-threatening pathologies and to retain this capability over time [35]. In that study, however, the study subjects were volunteers, which may have increased the students’ willingness and abilities to learn the POCUS basics.

### Prerequisites for implementing a treat-and-release strategy

The i-STAT alinity operates only if the internal temperature is within 16 to 30 °C [19]. We observed technical failures due to low operating temperature during the cold winter months of study. This forced us to develop simple warming systems in the ambulances. There were no reported technical or operational problems with QuickReady Go and LUMIFY during the project period.

The objective of this study was not to assign the EMTs and PMs the overall competences to investigate the patients and on their own to make the decision to release the patient at the scene or to admit the patient. Thus, to establish similar concepts like ours, where medical decisions were made using telemedicine (i.e. tele-counselling, or real-time tele-ultrasound), it is imperative that the internet connection is sufficient in all of the catchment area.

The inclusion of patients in the study was dependent on EMTs or PMs in the field, and we acknowledge that prehospital personnel sometimes work under time-dependent conditions which might have played a factor for missed inclusion of patients fulfilling the inclusion criteria.

A total of 28 patients did not fulfil the protocol as they expressed their wish to be admitted to hospital either before, during or after inclusion into the project. A major factor may be that patients feel safer when treated in a hospital or having physical contact with a physician. Future studies are warranted in this respect.

The present outpatient treatment strategy for AE-COPD patients was applicable due to POCT. POCT, when used in appropriate scenarios, could be a useful tool to minimize the time-to-treatment initiation by providing immediate information to healthcare professionals about the condition of the patient and improve patient outcomes [36]. Numerous reports highlight decreases in turnaround times for test results with POCT in an emergency setting [37, 38]. Also, the use of POCUS by emergency physicians has increased in the past decades, and it is now an essential diagnostic tool routinely used in EDs.

Even though POC-devices are portable and can be carried to the patient, the devices do not necessarily come with the same expertise as a laboratory physician would provide. Furthermore, the quality of the measurements may pose a problem. Although newer of POC-equipment have inbuilt quality control, it is vital to maintain internal and external controls of the devices. We thus suggest that future POC-systems are implemented with the assistance of the departments of laboratory medicine.

### Strengths of the study

In our study, the assignment of the POCUS and blood test competences were pragmatically based on the rotas of the participating EMTs and PMs. Thus, we did not only include prehospital personnel with specific interests and specific competences in acquiring new knowledge but tested the concept in a large scale.

In the Danish prehospital setting a certified EMT have almost 2½ to 4 years of education [14]. It is possible that other prehospital personnel with a different educational background will affect the outcome in other prehospital systems.

### Limitations of the study

Despite 771 patients were screened as requiring an ambulance because of respiratory complaints, only 81 were potential candidates for the study. Although previously diagnosed with COPD, a large proportion of the patients initially screened had called the emergency medical services for complaints that could not solely be attributed to an exacerbation of COPD. Previously, we have shown that the prehospital clinical characteristics of COPD patients does not allow for prognostication as patients with COPD constitute a very disperse group [7]. As our study clearly shows, there are thus limitations to the level of ambitions regarding treat-and-release of patients solely suffering from COPD.

Another important limitation in this study is that only half of the patients that were assessed as potential candidates for the treat-and-release strategy were included in the study. Fifteen of the patients that were offered participation in the study declined participation before the investigations were carried out, while 20 of the potentially eligible patients were not included without any documented reason for the EMTs or PMs not to include them. This may be caused by the pragmatic principle of the study. It is possible that some of the EMTs or PMs were not comfortable with the increased level of competences and thus omitted to implement their newly assigned competences.

## Conclusion

It is feasible for prehospital EMTs or PMs to perform prehospital point of care ultrasound and laboratory testing among patients with acute exacerbations of COPD. This may enable a treat-and-release strategy in selected COPD patients, thus reducing the number of emergency department visits and short hospital admissions. The concept of adding advanced competences to the curriculum of EMTs and PMs, however, may show promise in other patient categories where point-of-care technology may add valuable knowledge to the patients’ condition.

## Data Availability

Anonymised data are available on reasonable request. Author SM should be contacted: Soeren.mikkelsen@rsyd.dk.
